# Ultrasonic Technique for Density Measurement of Liquids in Extreme Conditions

**DOI:** 10.3390/s150819393

**Published:** 2015-08-07

**Authors:** Rymantas Kazys, Reimondas Sliteris, Regina Rekuviene, Egidijus Zukauskas, Liudas Mazeika

**Affiliations:** Ultrasound Research Institute, Kaunas University of Technology, Barsausko st. 59, Kaunas LT-51368, Lithuania; E-Mails: rymantas.kazys@ktu.lt (R.K.); reimondas.sliteris@ktu.lt (R.S.); e.zukauskas@ktu.lt (E.Z.); liudas.mazeika@ktu.lt (L.M.)

**Keywords:** ultrasonic measurements, density, extreme conditions

## Abstract

An ultrasonic technique, invariant to temperature changes, for a density measurement of different liquids under *in situ* extreme conditions is presented. The influence of geometry and material parameters of the measurement system (transducer, waveguide, matching layer) on measurement accuracy and reliability is analyzed theoretically along with experimental results. The proposed method is based on measurement of the amplitude of the ultrasonic wave, reflected from the interface of the solid/liquid medium under investigation. In order to enhance sensitivity, the use of a quarter wavelength acoustic matching layer is proposed. Therefore, the sensitivity of the measurement system increases significantly. Density measurements quite often must be performed in extreme conditions at high temperature (up to 220 °C) and high pressure. In this case, metal waveguides between piezoelectric transducer and the measured liquid are used in order to protect the conventional transducer from the influence of high temperature and to avoid depolarization. The presented ultrasonic density measurement technique is suitable for density measurement in different materials, including liquids and polymer melts in extreme conditions. A new calibration algorithm was proposed. The metrological evaluation of the measurement method was performed. The expanded measurement uncertainty U_ρ_ = 7.4 × 10^−3^ g/cm^3^ (1%).

## 1. Introduction

Continuous monitoring of liquid or molten material parameters is a fundamental requirement for process control [1]. Moreover, temperature, pressure and other process parameters, such as level, flow rate, density and concentration, are of a special interest [2,3]. An important mechanical property of liquids is density. This is a fundamental parameter for the quality of the final product and a very significant factor affecting the production cost and profitability of the manufacturing process [4,5]. The density of various liquid substances usually is measured in laboratory conditions. For this purpose, well-known density measurement instruments, such as pycnometers, aerometers or hydrometers, are often used, but they are not suitable for process control applications [6]. Those standard “off-line” laboratory assessments may be misleading, particularly if the molecular features of the liquid change during the test [7,8]. The time delay between collecting the samples and obtaining the results can last from several minutes to several hours, which is not suitable for manufacturing processes [9,10].

In-process measurements may be on-line or in-line [11,12]. On-line measurements require the liquid to be sampled from the main process. In-line measurement aims to obtain data from the main process flow without disturbing this flow. There are many advanced techniques suitable for in-line measurements: near-infra-red, spectroscopic [10], X-ray absorption [13], electrical [14] and ultrasonic [15,16,17,18,19,20]. In the last case, different types of ultrasonic waves are used: longitudinal [9], shear [14], Love [16,17] and torsional waves [18,19,20]. Each of them has its own advantages and disadvantages. The spectroscopic measurement method is accurate and reliable, but expensive. The dielectric density measurement method usually is combined with other measurement methods, e.g., ultrasonic [14]. The ultrasonic measurement methods are accurate, reliable, but in most cases, can be applied for density measurements of different liquids only at temperatures close to room temperature [16,18].

In general, there are two main ultrasonic methods that are proposed for density measurements of various liquids: the transmission method [10] and the pulse-echo method [21,22,23]. In the first case, the method is based on the measurement of the ultrasonic wave transmitted through the solid/liquid and liquid/solid interfaces under test, and the transmission coefficient *T* is calculated. The pulse-echo method is based on the measurement of the ultrasonic wave reflected from the interface solid/liquid medium under test, and the reflection coefficient *R* is calculated. The density of the liquid is found from the measured transmission or reflection coefficients. Measurements in the transmission mode in most cases are very problematic due to the specific geometry of the measurement system and the complicated access to the liquid under measurement. Therefore, for in-line density measurements, the pulse-echo method is more suitable. However, until now, such density measurements in various liquids mainly were performed at a room temperature (*T* = 21 °C) [22,23]. In this case, temperature changes do not influence the sensitivity of the measurement system, because the temperature is constant.

Our previous work revealed that serious problems arise when the density measurements must be carried out during the manufacturing processes at high temperature (180–250 °C) and high pressure (1–10 MPa) [24]. The conventional ultrasonic transducers used for the generation and reception of ultrasonic waves cannot withstand high temperatures and pressure. In order to protect the piezoelectric elements from the influence of high temperature, special waveguide transducers with a relatively low thermal conductivity can be used [25,26,27,28,29,30].

For density measurements in extreme conditions, we have proposed the measurement method in which the ultrasonic signals reflected from the tip of the waveguide contacting the measured liquid are exploited [31]. This method is suitable for on-line density measurements. In this work, we mainly focused on the density measurement technique itself, but the technique’s performance in extreme conditions was not investigated in detail.

The objective of the presented work was the implementation and detailed investigation of the performance of the developed technique in extreme conditions, including high variable temperatures. Special attention was paid to the measurement of the density of melted polymers during a manufacturing process, during which high variable temperatures and pressure exist.

## 2. Theory, Analysis and Modelling Results

### 2.1. The Principle of the Measurement Method

**Figure 1:**
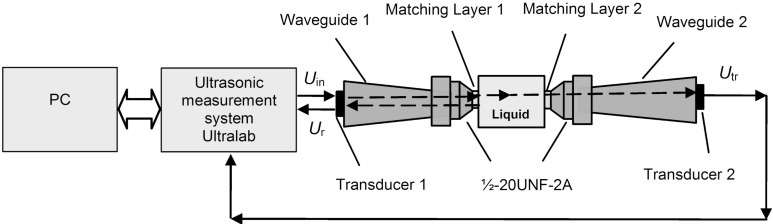
Ultrasonic density measurement system.

The proposed ultrasonic density measurement method is based on the measurement of the ultrasonic wave reflected from the interface solid/liquid medium under test. The structure of the proposed density measurement system is presented in Figure 1. The ultrasonic measurement system consists of Ultrasonic Transducer 1 (pulser/receiver), Ultrasonic Transducer 2 (receiver), two waveguides, 1 and 2, with acoustic impedance *Z*_1_, two λ/4 acoustic impedance matching layers, 1 and 2, with acoustic impedance *Z*_2_, and the measured liquid with acoustic impedance *Z*_3_. Selection of the materials for matching layers is presented in Section 2.1. Calculation of the acoustic impedance of the matching layer is based on a matrix model [32]. The measurements under *in situ* extreme conditions usually must be carried out through a relatively narrow access standard port (e.g., ½-20UNF-2A see Unified Screw Thread standard). In order to separate transmitted and reflected signals in the time domain, pulsed ultrasonic signals are used. Ultrasonic Transducer 1 with a piezoelectric element (Pz 29) generates an acoustic pulse that travels through Waveguide 1, Acoustic Matching Layer 1 (*Z*_2_) and reaches the liquid medium, the density of which is measured (*Z*_3_). The ultrasonic pulse wave generated by Ultrasonic Transducer 1 is transmitted through the measured liquid and received by Transducer 2. The transmitted pulse through the interface matching layer-liquid *U*_tr_ is exploited for the measurement of the ultrasound velocity *c*_3_ in the measured liquid. Another part of the ultrasound wave is reflected from the solid/liquid interface due to a mismatch of acoustic impedances *Z*_2_ and *Z*_3_ between the waveguide and measurement liquid. From the received signal *U*_r_, only the pulse reflected by the tip of the waveguide is selected in the time domain and is used for the determination of the liquid density ρ_3_.

The proposed density measurement method is based on the measurement of the reflection coefficient *R*_3_ of the ultrasonic wave from the solid/liquid interface. Please note that the presented analysis of the measurement method is based on an assumption of plane ultrasonic waves, e.g., the one-dimensional (1D) approach. The reflection coefficient *R*_3_ can be found from the amplitudes of the incident and reflected waves:

(1) $$R_{3}=\frac{U_{in}}{U_{r}}$$

where *U*_r_ is the amplitude of the ultrasonic signal reflected from the solid/liquid interface and *U*_in_ is the amplitude of the incident signal.

In order to enhance the sensitivity of the measurements, the tip of Waveguide 1 is coated by a λ/4 matching layer. Then, the reflection coefficient *R*_3_(ω) is frequency dependent and can be found from the following equation [29]:

(2) $$R_{3}(\omega )=\frac{Z_{IN}(\omega )-Z_{1}}{Z_{IN}(\omega )+Z_{1}}$$

where the *Z*_IN_(ω) is the input acoustic impedance of the matching layer. The calculation of the *Z*_IN_(ω) is based on a matrix model [32]. At the frequency ω = ω_0_ (ω = 2π*f*), at which the thickness of the matching layer is *l =* λ_0_/4, the input acoustic impedance *Z*_IN_(ω) is given by [29]:

(3) $$Z_{IN}(\omega_{0})=\frac{Z_{2}^{2}}{Z_{3}}$$

where *Z*_2_ is the acoustic impedance of the matching layer material, *Z*_3_ is the acoustic impedance of the liquid medium, λ_0_ is the wavelength in the matching layer λ_0_ = *c*_2_/*f*_0_, *c*_2_ is the ultrasound wave velocity in the matching layer and *f*_0_ is the resonant frequency.

The acoustic impedance of the measured liquid *Z*_3_ can be found from Equations (2) and (3) [29]:

(4) $$Z_{3}=\frac{Z_{2}^{2}\cdot \left(1-R_{3}(\omega_{0})\right)}{Z_{1}\cdot (1+R_{3}(\omega_{0}))}$$

The acoustic impedances *Z*_1_ and *Z*_2_ must be known in advance. In general, properly selecting the material of the matching layer, it is possible to enhance the sensitivity of the density measurement significantly [24]. The maximal sensitivity is obtained when the acoustic impedance of the matching layer *Z*_2_ is close to $Z_{2}\approx \sqrt{Z_{1}\cdot Z_{3}}$, where *Z*_1_ is the acoustic impedance of the waveguide material and *Z*_3_ is the acoustic impedance of the measured liquid medium. In our case, the impedance *Z*_2_ must be intermediate between the acoustic impedances of the waveguide *Z*_1_ and the measured liquid *Z*_3_ [33,34,35]. Steel and titanium were proposed as materials for the waveguides (Section 2.1). In this case, the λ/4 acoustic matching layer can be made from following materials: compound Duralco 4703, plastic PBI (polybenzimidazole), aluminum powder and glass enamel. The acoustic parameters of those materials are presented in Table 1.

In order to determine the absolute value of the reflection coefficient *R*_3_(ω_0_), the amplitudes of the incident *U*_in_ and reflected *U*_r_ waves at the frequency *f*_0_ are necessary. Taking into account that for measurements, pulse-type signals are used, those amplitudes are found from the spectra of the corresponding signals. The amplitude of the reflected signal *U*_r_ at the frequency *f = f*_0_ is given by:

(5) $$U_{r}\left(f_{0}\right)={\left|FFT\left[U_{l}(t)\right]\right|}_{|f=f_{0}}$$

where *U*_l_(*t*) is the signal recorded by Transducer 1 in a reception mode.

The amplitude of the incident wave *U*_in_ cannot be found in such simple way, because in this case, a separate receiver of the incident ultrasonic wave should be necessary. Instead, this measurement is replaced by an additional measurement of the reflected signal *U*_rw_ from distilled water, the acoustic parameters of which are well known in a wide temperature range. Then, the reflection coefficient *R*_3_(*f*_0_) is found from the ratio of the amplitudes of the signals reflected from the measured liquid and the distilled water:

(6) $$R_{3}(f_{0})=\frac{U_{r}}{U_{rw}}\cdot R_{3w}(f_{0})$$

where $R_{3w}(f_{0})$ is the reflection coefficient in the case of distilled water, which is found during the calibration procedure. Then, the acoustic impedance of the measured liquid *Z*_3_ is found from Equation (4).

Please note that the acoustic impedance of the measured liquid *Z*_3_ depends on the temperature *T*:

(7) $$Z_{3}(T)=\rho_{3}(T)\cdot c_{3}(T)$$

**Figure 2:**
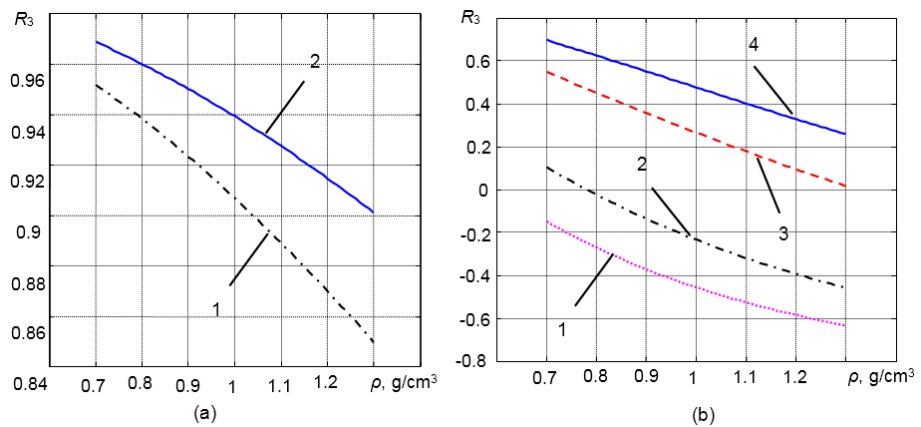
The reflection coefficient *R*_3_ *versus* the density of the medium ρ_3_ contacting the tip of the different waveguides (estimated from modelling): (**a**) without matching layer: 1, titanium waveguide; 2, steel waveguide; (**b**) with the λ/4 matching layers: 1, titanium waveguide with the polybenzimidazole (PBI) matching layer; 2, titanium waveguide with the Duralco matching layer; 3, steel waveguide with the aluminum powder matching layer; 4, steel waveguide with the glass enamel matching layer.

The acoustic impedance of the measured liquid *Z*_3_ depends on the density ρ_3_ and the ultrasound wave velocity *c*_3_ at the measurement temperature *T*. In order to eliminate the influence of the temperature *T*, the ultrasound wave velocity ${\hat{c}}_{3}$ in the liquid medium is measured in the transmission mode. Taking into account Equation (7), the measured density of the liquid is ${\hat{\rho }}_{3}(T)$, obtained from Equation (8):

(8) $${\hat{\rho }}_{3}(T)=\frac{Z_{3}(T)}{{\hat{c}}_{3}(T)}$$

where **^** denotes the measured values at the given temperature. As was mentioned above, the sensitivity of the measurement system to density variations is directly related to variations of the reflection coefficient *R*_3_. In order to investigate how the reflection coefficient *R*_3_ depends on the density of the liquid medium ρ_3_, 1D analytical modelling was performed. In the case of solid waveguides made of steel or titanium, those variations are quite small (Figure 2a). That is due to a very significant mismatch of the acoustic impedances of steel (*Z*_1_ = 44.5 MRayl) or titanium (*Z*_1_ = 28.5 MRayl) and the measured liquids *Z*_3_ = 0.6–1.8 MRayl.

In order to enhance the sensitivity of the measurement system, transformation of the acoustic impedance of the liquid *Z*_3_ by means of the λ/4 impedance matching layer has been proposed. The matching layers can be made of different materials. Those materials must meet the following requirements: the acoustic impedance of the λ/4 matching layer must be intermediate between the waveguide transducer and the measured liquid, resistant to high temperature and pressure and possess stable acoustic properties in a given temperature range. In general, it is not easy to find the materials corresponding to the requirements mentioned above. Some materials that can be used for matching layers are the following: compound Duralco 4703, plastic PBI (polybenzimidazole), aluminum powder and glass enamel. The properties of those materials are presented in Table 1.

**Table 1:** Properties of the materials.

Materials	Maximum Operation Temperature *T*_max_, °C	Acoustic Impedances *Z*, MRayl
Compound Duralco	250	4.95
PBI	310	3.85
Aluminum powder	580	10.34
Glass enamel	1500	13.22

The reflection coefficients *R*_3_ *versus* the density of the medium ρ_3_ contacting the tip of different waveguides with the λ/4 matching layers (listed in Table 1) are presented in Figure 2b. From the results shown in Figure 2a,b, it follows that variations of the reflection coefficient *R*_3_ due to changes of the density of the medium under investigation in the case of the λ/4 matching layer are much bigger than in the case of the waveguide without a matching layer. Therefore, the sensitivity of the measurement system increases significantly. For example, in the case of the steel waveguide with the aluminum powder matching layer, the reflection coefficient is changing in the range *R*_3_ = 0.05–0.55, when the density of the liquid changes in the range ρ = 0.7–1.3 g/cm^3^.

### 2.2. The Design of the Ultrasonic Transducer with a Waveguide

The main part of the proposed density measurement system is the ultrasonic transducer with a waveguide, which protects a piezoelectric element from the influence of high temperature and pressure. The geometry and dimensions of the waveguide must be suitable to fulfil such requirements.

**Figure 3:**
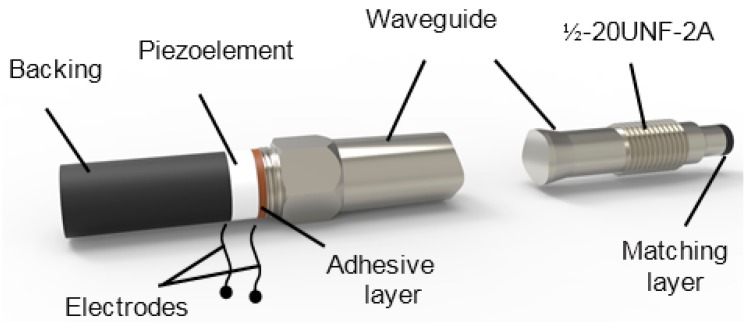
Ultrasonic transducer with a waveguide.

A general view of the designed ultrasonic transducer with a metallic waveguide is shown in Figure 3. The waveguide is screwed via standard port ½-20UNF-2A into a wall of the high pressure pipe in which the measured liquid is flowing. Such a design enables on-line measurements.

Lateral dimensions of the waveguide, first of all, are defined by the half-inch standard port, and the diameter of the waveguide tip contacting the measured liquid is 8 mm. The tip of the waveguide is coated by a λ/4 matching layer, and its temperature in many cases may reach 200–250 °C. The length of the waveguide must be long enough in order to reduce the temperature from 200 °C to the temperature lower than the allowed operation temperature of the piezoelectric element made of piezoceramics Pz 29 and the adhesive layer between this element and the waveguide. The Curie temperature of Pz 29 material is *T*_C_ = 235 °C, e.g., quite high, but the allowed temperature of the adhesive layer used to bond the piezoelectric element to the waveguide can be <100 °C.

**Figure 4:**
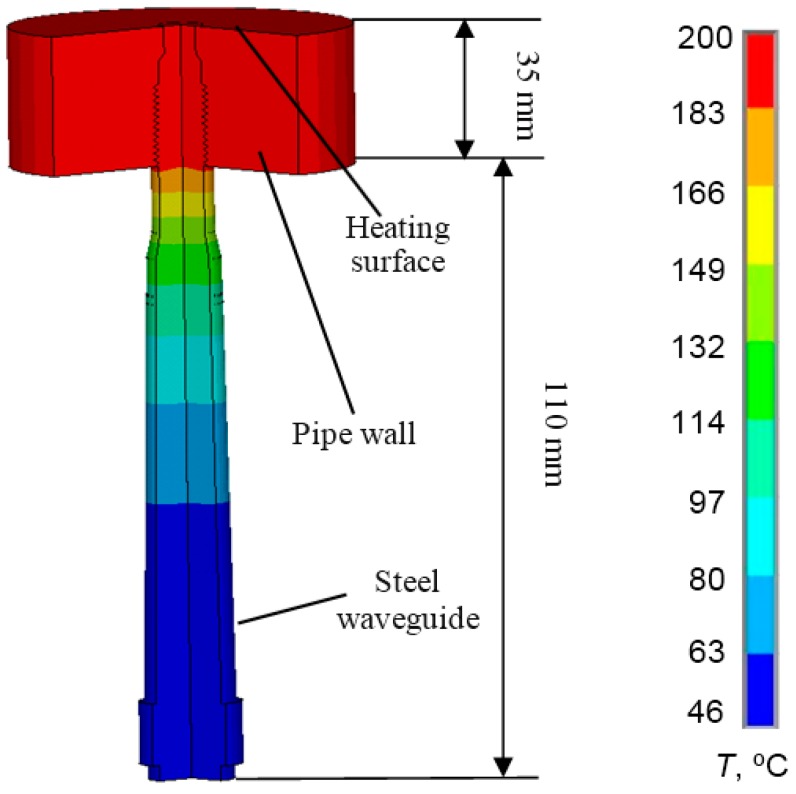
Temperature distribution in the AISI 316 steel waveguide.

**Figure 5:**
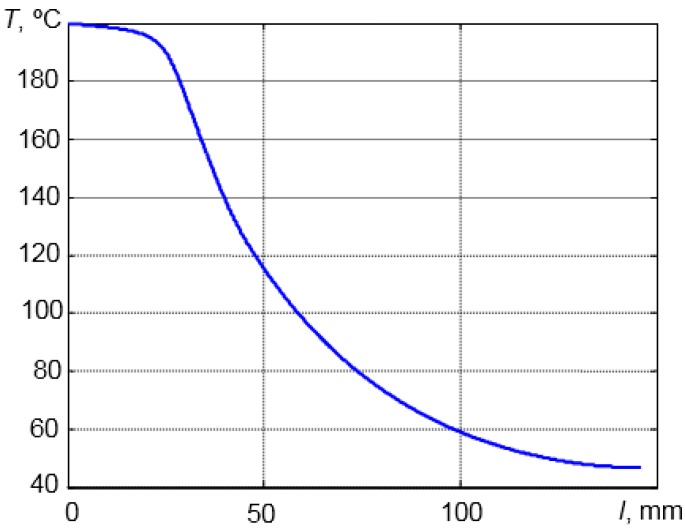
Temperature distribution along the center line of the waveguide.

In order to determine the suitable length of the waveguide temperature distribution in the stainless steel, the AISI 316 waveguide was calculated using the ANSYS finite element code. The simulation results in the steel waveguide with the λ/4 aluminum powder matching layer are presented in Figure 4 and Figure 5.

The modelling was carried out using 2D elements and the axial symmetry option. It was assumed that the temperature of surrounding air was *T*_air_ = 20 °C and that the temperature of the heated surface was 200 °C. The end of the waveguide is fixed in the steel wall and heated by a hot liquid. The piezoelectric element was fixed to the opposite end of the waveguide (Figure 4). The thermal conductivity of AISI 316 stainless steel is 16.2 W/m∙K at 100 °C and 21.4 W/m∙K at 500 °C. According to the literature, the heat transfer coefficient *h* in the case of a free convection is 6–30 W/m^2^∙K when the surrounding medium is air [36]. During 2D FE modelling, we have assumed that the heat transfer coefficient is 20 W/m^2^·K.

From the simulation results (Figure 4 and Figure 5), it follows that even when the tip of the waveguide is heated up to the *T* = 200 °C, the temperature of the opposite end of the waveguide to which the transducer was bonded does not exceed *T* = 50 °C. Therefore, the length of the waveguide *l* = 145 mm is sufficient for high-temperature measurements.

As was mentioned above, the density measurement is performed in pulse-echo and transmission modes. The accuracy of the measurements in this case depends on the frequency response of the measurement system. The frequency response of the whole waveguide transducer depends on the dimensions and materials of all constitutive elements, such as the piezoelectric element, the adhesive layer, the λ/4 matching layer, the backing and the waveguide (Figure 3). Therefore, the influence of the separate elements of the multilayered system on the transducer frequency responses must be found.

**Figure 6:**
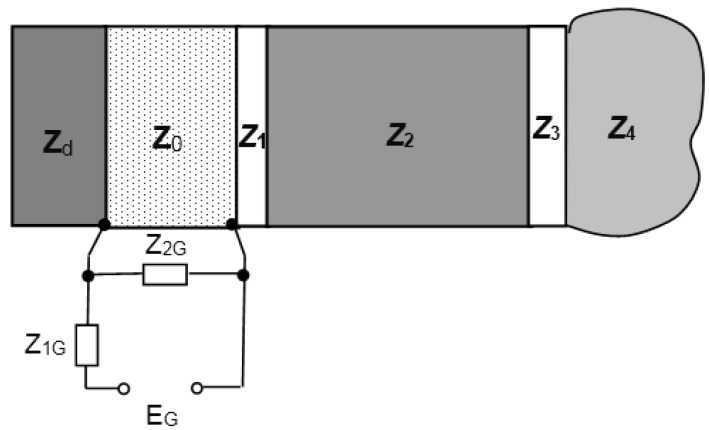
The mathematical model of the piezoelectric transducer with waveguide. Acoustic impedances of the elements: *Z*_d_, damping; *Z*_0_, piezo element; *Z*_1_, adhesive layer; *Z*_2_, waveguide; *Z*_3_, matching layer; *Z*_4_, measured medium; *Z*_1G_,_2G_, electrical impedances.

In order to find the requirements for the adhesive layer, the calculation of the frequency responses based on a matrix model [31] was performed. The 1D model of the ultrasonic transducer is shown in Figure 6. The calculations were performed in the frequency range *f* = 1–8 MHz. It was assumed that the adhesive layer is epoxy glue (Table 2).

**Table 2:** Properties of the adhesive layer.

Materials	Density ρ, kg/m^3^	Ultrasound Wave Velocity *c*_L_, m/s	Acoustic Impedance Z, MRayl
Glue (epoxy)	1428	2800	4

**Figure 7:**
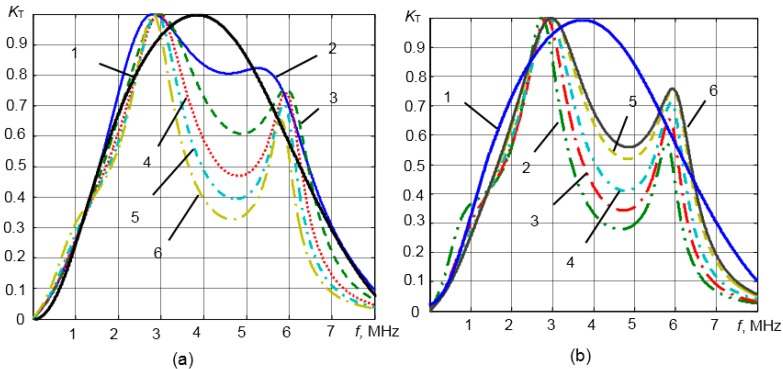
(**a**) Calculated influence of the adhesive layer thickness *d*_1_ on transducer frequency responses: 1, single-layer piezoelectric transducer; 2, 0 μm; 3, 10 μm; 4, 20 μm; 5, 30 μm; 6, 50 μm; (**b**) Influence of the adhesive layer acoustic impedance *Z*_1_ on transducer frequency responses: 1, single-layer piezoelectric transducer; 2, 2 MRayl; 3, 3 MRayl; 4, 4 MRayl; 5, 6 MRayl; 6, 7 MRayl.

The influence of the adhesive layer acoustic impedance and thickness on the transducer frequency response is presented in Figure 7a,b. It was assumed that the multi-layered piezoelectric transducer with the steel waveguide is loaded by the liquid acoustic impedance, which is *Z*_4_ = 1.8 MRayl. The resonance frequency of the piezoelectric element was 3.3 MHz. The frequency response of a single-layer piezoelectric element without the adhesive layer is presented in Figure 7a,b by Curve 1. *K*_Tnorm_(*f*) = *K*_T_(*f*)/*K*_max_(*f*) is the normalized transfer function of the multi-layered transducer in the transmission mode; *K*_T_(*f*) = *p*(*f*)/*U*_in_(*f*), *p*(*f*) is the acoustic pressure in the measured liquid; *U*_in_(*f*) is the electric voltage at the input of the piezoelectric element; and *K*_max_(*f*) is the maximal value of the transfer function.

The frequency responses of the multi-layered transducer with the adhesive layer of different thicknesses in the range *d* = 10–50 μm are shown in Figure 7a. The acoustic impedance of the adhesive layer was *Z*_1_ = 4 MRayl. From the results presented, it follows that when the thickness of the adhesive layer is increasing, the non-uniformity of the frequency response is increasing, as well. For operation in pulse mode, the frequency response with two clear resonance peaks is not attractive, because in this case, distortions of the pulse waveforms start to be noticeable. Therefore, the thickness of the adhesive layer must be as small as possible, e.g., ≤10 μm.

The calculated influence of the acoustic impedance of the adhesive layer on the frequency responses is shown Figure 7b. The thickness of the adhesive layer was 10 μm. The acoustic impedances were changed in the range *Z*_1_ = 2–7 MRayl. From the results presented in Figure 7b, it follows that the acoustic impedance of the adhesive layer also significantly influences the transducer frequency responses. The frequency responses also possess two resonance peaks instead of one. When the acoustic impedance of the adhesive layer is decreasing, the non-uniformity of frequency responses is increasing. Therefore, the acoustic impedance of the adhesive layer should be *Z*_1_ ≥ 6–7 MRayl.

### 2.3. Investigation of High Temperature Influence on the Acoustic Parameters of the Materials (1D Model)

As we mentioned above, the density measurements in different liquids quite often must be performed at high temperature and pressure. Additional problems arise when the temperature is not constant and changes for a time. In this case, the density measurements can be very problematic. A high temperature has a significant influence on the transmission of the ultrasonic wave from the ultrasonic waveguide transducer to the environment under investigation. The biggest influence is due to the temperature-dependent ultrasound wave velocity *c*. Therefore, the selection of the appropriate materials for the ultrasonic measurement system is very important [37].

As waveguide materials suitable for operation in a wide temperature range, the stainless steel AISI 316 and titanium can be used [38]. The thermal conductivity of those materials is relatively low: for titanium Grade 2, it is 16.7 W·m^−1^·K^−1^, and for the stainless steel AISI 316 21.9 W·m^−1^·K^−1^, respectively; therefore they, are suitable as thermal insulators between the high temperature zone and a piezoelectric element.

The relationship between the ultrasound wave velocity *c* and the temperature change Δ*T* in solids can be approximated by a linear dependence:

(9) $$c(T)=c_{0}-T_{K}\cdot \Delta T$$

where *c*(*T*) is the ultrasound wave velocity in a material at the given temperature *T*, *c*_0_ is the ultrasound wave velocity at room temperature (*T*_0_ = 21 °C), Δ*T* = *T* − *T*_0_ is the temperature variation in a material and *T*_K_ = (*c*_0_ − *c*(*T*))/Δ*T* is the ultrasound velocity temperature coefficient.

The temperature coefficient *T*_K_ of the material is a very important parameter describing the ultrasound wave velocity changes at different temperatures. Lower values of the *T*_K_ give lower ultrasound wave velocity changes. The ultrasound velocity temperature coefficients of the waveguide materials, such as a titanium and stainless steel AISI 316, are well known. In the stainless steel AISI 316, for longitudinal waves, the temperature coefficient is *T*_K_ = −0.7 m/s per Celsius degree [39] and in titanium *T*_K_ = 2.5 m/s per Celsius degree [40].

The requirements for materials of the matching layers were formulated in the previous paragraph. Several different materials were proposed and investigated as matching layers (Table 1). The performance of the compound Duralco 4703, the plastic PBI (polybenzimidazole) and the pressed aluminum powder as matching layers has not been investigated yet. Therefore, measurements were carried out of the ultrasound longitudinal wave velocity *c*_L_ at different temperatures, and the ultrasound velocity temperature coefficient *T*_K_ was calculated. The results of measurements denoted by * are presented in Table 3. The temperature coefficients of the aluminum powder is *T*_K_ = 1.3 m/s, the Duralco *T*_K_ = 4.9 m/s and the PBI *T*_K_ = 2.1 m/s.

From the results obtained, it follows that the most unstable material in a wide temperature range is the compound Duralco 4703; the most stable is the pressed aluminum powder. The acoustic properties of the glass enamel (ρ = 2.15 g/cm^3^; *Z* = 13 MRayl) are similar to the properties of the fused silica (ρ = 2.2 g/cm^3^; *Z* = 13 MRayl). According to the measurements of the ultrasound wave velocities and acoustic impedances of the glass enamel at elevated temperatures, the temperature coefficient of the glass enamel is *T*_K_ = 0.7 m/s/°C, e.g., quite low [41,42].

**Table 3:** Ultrasound longitudinal wave velocities *c*_L_ for materials at elevated temperatures.

	Material					
	Steel	Titanium	Aluminum Powder *	Duralco *	PBI *	Glass Enamel
*T*, °C	Ultrasound Wave Velocity *c*_L_, m/s					
20	5740	6203	4700	2615	2970	5950
100	5681	6003	4593	2215	2790	6005
150	5649	5878	4565	1980	2685	6040
200	5614	5753	4510	1720	2575	6075

* Measured absolute values of the ultrasound longitudinal wave velocity.

**Figure 8:**
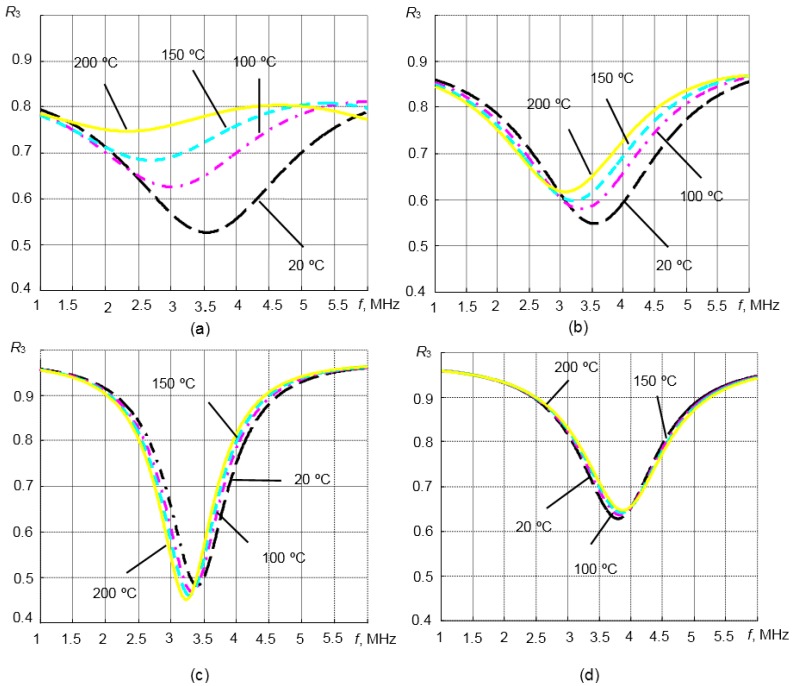
The reflection coefficient *R*_3_ from the tip of the waveguide with the λ/4 matching layer *versus* that of a frequency at different temperatures. (**a**) Titanium waveguide with the Duralco matching layer; (**b**) titanium waveguide with the PBI matching layer; (**c**) steel waveguide with the glass enamel matching layer; (**d**) steel waveguide with the aluminum powder matching layer.

In order to estimate the performance of the matching layers made of those materials, listed in Table 3, the frequency responses of the reflection coefficient *R*_3_ were calculated (Equation (2)) in the temperature range 20–200 °C. The calculations were performed using the 1D matrix model for titanium and steel waveguides. The load in this case was liquid with the acoustic impedance *Z*_3_ = 1.8 MRayl. Such acoustic impedance corresponds quite well to the acoustic parameters of most liquids used in many fields of industry [1,23,24]. The results are presented in Figure 8a–d. As was possible to expect, the biggest influence of temperature variations is observed in the case of the titanium waveguide with compound Duralco 4703 and PBI matching layers (Figure 8a,b). The temperature has no essential influence on the reflection coefficients *R*_3_ values in the case of the steel waveguide coated by the pressed aluminum powder and glass enamel λ/4 matching layers (Figure 8c,d). Please note that in the case of glass enamel, the reflection coefficient *R*_3_ is decreasing with increasing temperature.

In our case, the steel waveguide with the λ/4 aluminum powder matching layer is more suitable for the high temperature measurements than a titanium waveguide. When the temperature is increasing in the range *T* = 20–200 °C, variations of the ultrasound wave velocity *c* in the steel waveguide with the λ/4 aluminum powder matching layer are insignificant, and changes of the reflection coefficient *R*_3_ are very small (Figure 8d). The reflection coefficient in this case at the resonance frequency is smallest *R*_3_ = 0.46 (*T* = 200 °C). Therefore, the steel waveguide with the λ/4 aluminum powder matching layer was selected for measurements at high temperatures.

### 2.4. Investigation of the Displacement Fields of the Ultrasonic Longitudinal Wave Propagating in the Tapered Geometry Waveguide Transducer (3D Model)

Our previous work revealed that the transducers with a buffer rod, a so-called waveguide-type transducer, can withstand high temperatures, pressures and corrosion attack, but measurements become more complicated due to multiple reflections inside the waveguide [32]. The presented previous analysis of the measurement method was based mainly on an assumption that plane ultrasonic waves propagate in transducer waveguides, e.g., it was based on the one-dimensional (1D) approach. However, in the case of waveguides with finite lateral dimensions, the situation is more complicated. Additional problems are caused by the requirement that the measurements *in situ* should be carried out through a relatively narrow access standard port ½-20UNF-2A. In this case, two questions arise: how close the 1D modelling results are to the real 3D situation and how the propagation of an ultrasound wave in the tapered waveguide is affected by the standard port (e.g., ½-20UNF-2A).

**Figure 9:**
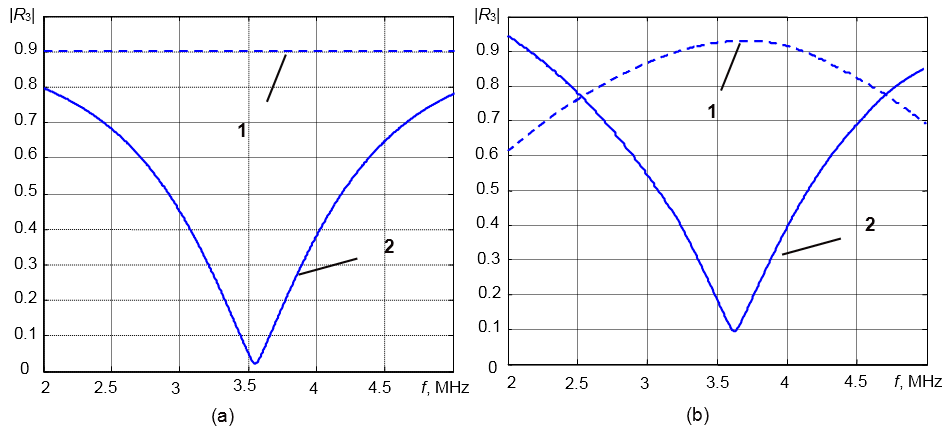
(**a**) Steel waveguide transducer reflection coefficient *R*_3_ *versus* frequency (1D model); (**b**) steel waveguide transducer reflection coefficient *R*_3_ *versus* frequency (3D model). 1, (dashed) without matching layer; 2, (line) with the λ/4 aluminum powder matching layer.

In order to answer those questions, the three-dimensional numerical modelling of the waveguide transducer with the λ/4 matching layer was performed and compared with the one-dimensional analytical model (Figure 9a,b).

In this case, the λ/4 matching layer was made of the pressed aluminum powder, the properties of which are given in Table 1. The load in this case was distilled water with acoustic impedance *Z*_3_ = 1.47 MRayl. From the results presented in Figure 9, it follows that variations of the reflection coefficient *R*_3_ with the matching layer due to changes of the acoustic impedance of the medium under investigation in both cases (1D, 3D) are much bigger (Figure 9a,b (Curve 2)) than in the case of the waveguide without a matching layer (Figure 9a,b (Curve 1)). The sensitivity of the measurement system increases significantly due to the matching layer. It is essential to notice that the results obtained by the 1D analytical model (Figure 9a) are quite close to the results obtained by the 3D numerical modelling (Figure 9b).

**Figure 10:**
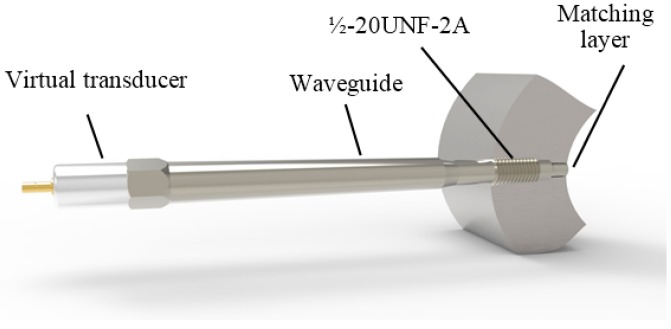
Graphical representation of the 3D model.

Graphical representation of the model used in 3D finite difference technique (Wave 3000) is presented in Figure 10. The diameter of the tip of the modelled ultrasonic transducer was 9 mm. The generation and reception of the ultrasonic waves in the model was performed by a virtual transducer (transparent for ultrasonic waves), which was operating in a pulse-echo mode and was placed on the wide end on the waveguide. The ultrasonic transducer generated a normal displacement. For excitation of the ultrasonic wave, the 3.3 MHz, three-period sine burst with the Gaussian envelope was used. The duration of the time step used in modelling was 14 ns. During modelling, the reflection coefficient *R*_3_ *versus* frequency was calculated.

**Figure 11:**
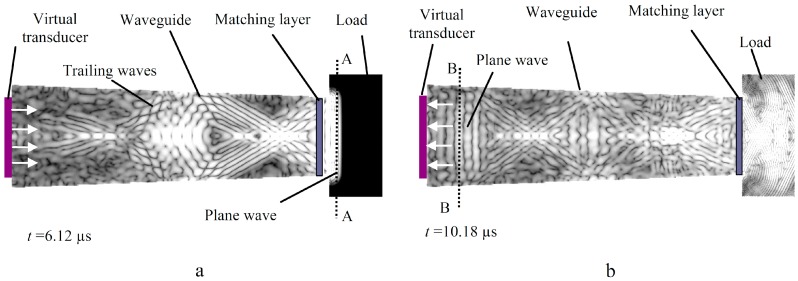
Displacement fields of the ultrasonic wave propagating in the tapered steel waveguides with the aluminum powder matching layer. (**a**,**b**) Cross-section *y*0*z* of the waveguide.

The displacement fields of the ultrasonic wave propagating in the tapered steel waveguide with the aluminum powder matching layer at different time instants were investigated. The results are presented in the *y*0*z* cross-section (Figure 11a,b) of the waveguide. In this case, a complex structure of the displacement fields is observed. Our investigations revealed that the longitudinal wave, which is used for the measurements, arrives first (Figure 11a). It is necessary to note that the longitudinal waves transmitted into a liquid load (Figure 11a, Cross-section A-A) and reflected back to the ultrasonic transducer (Figure 11b, Cross-section B-B) are very close to a plane wave. Therefore, no noticeable diffraction errors during measurements were observed.

Multiple trailing waves are travelling behind the longitudinal wave (Figure 11b). Such signals are unwanted, because they can overlap with the wave of interest and cause significant measurement errors. Therefore, the signals used in measurements were selected in the time domain (discussed in Section 3.1) in order to avoid the influence of the trailing waves.

It is essential to note that the length of the waveguide strongly affects the trailing waves. Too long of a waveguide may cause additional reflections from the waveguide boundaries. On the other hand, as was mentioned in Section 2.2, too short of a waveguide does not enable reducing the temperature at the ultrasonic transducer to an acceptable level below 100 °C. Therefore, the trade-off between those two contradictory requirements gives the optimal waveguide length *l* = 110 mm. Such a waveguide was selected for density measurements in different liquids in extreme conditions.

## 3. Experiments and Discussions

### 3.1. Calibration and Verification

**Figure 12:**
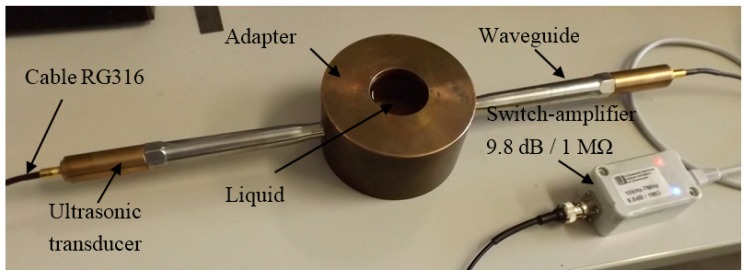
The calibration setup.

The proposed density measurement technique was calibrated under laboratory conditions using distilled water. The distilled water was used for the calibration procedure, because the temperature dependences of the ultrasound wave velocity *c* and density ρ of this liquid are well known with a high accuracy in a wide temperature range. The objective of the calibration procedure was to determine the static characteristic of the developed density measurement system relating the measured amplitudes *U*_rw_ and *U*_r_ of the signals reflected from the tip of the waveguide, loaded by the distilled water and other liquids to their densities. The calibration setup is shown in Figure 12. The measurement system Ultralab was developed and manufactured at Ultrasound Research Institute (Kaunas University of Technology). The temperature of the distilled water was measured by the K-type thermocouple and was *T_W_* = 19 °C.

At first, the distance between the tips of Waveguides 1 and 2 was measured. The mechanically-measured inner diameter of the adapter was *D =* 38 mm. However, the surfaces of the tips of the waveguides do not coincide exactly with the inner surface of the adapter. Therefore, in order to get a more accurate value of the distance between the waveguides, an additional measurement using the distilled water was performed in the through transmission mode. The time delay of the ultrasonic pulse τ_w_(*T*) in the distilled water at the known temperature *T* was measured. Then, taking into account the known ultrasound velocity *c*_w_(*T*) at the temperature *T*, the distance between the tips of Waveguides 1 and 2 can be found from the expression:

(10) $$l_{k}=c_{w}(T)\cdot \tau_{w}(T)$$

**Figure 13:**
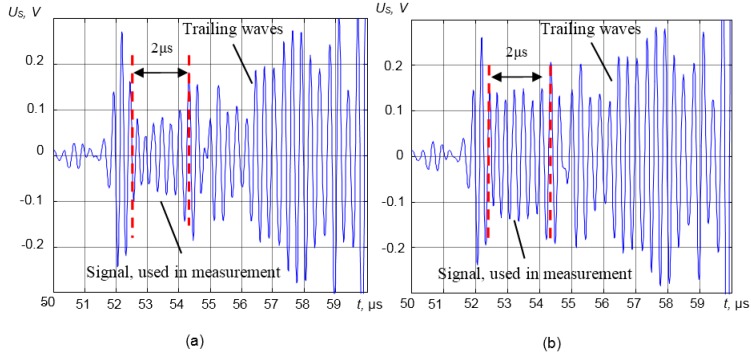
Time diagrams of the measured signals reflected by the interface waveguide-liquid and received by the ultrasonic transducer in the case of the steel waveguide with the aluminum powder matching layer with different loads: (**a**) distilled water; (**b**) ethyl ether.

The distance between the transducers measured in such way was *l* = 38.25 mm. The density measurement technique was verified under laboratory conditions with various liquids densities for which they are known or were measured: ethyl alcohol, ethyl ether and different concentrations of sugar water solutions. The densities of those liquids are in the range ρ = 0.7–1.4 g/cm^3^, and they are close to the densities of most liquids used in industry. For density measurements, the signals reflected from the tip of Waveguide 1 are exploited. As was mentioned above, the so-called trailing waves may overlap with the wave of interest and cause measurement uncertainty. Those trailing waves can arise due to the waveguide geometry and the standard port ½-20UNF-2A (Figure 3). The time diagrams of the measured signals reflected by the interface waveguide-liquid and received by the ultrasonic transducer in the case of the steel waveguide with the aluminum powder matching layer loaded by the distilled water and ethyl ether are presented in Figure 13a,b. The number of performed experiments with distilled water, ethyl ether, ethyl alcohol and different concentration sugar water solutions was *N* = 100. The signals reflected from the end of the waveguide are very complicated. However, in all cases, the first part of the reflected signal (Figure 13) between 50 μs and 52.5 μs is the reflection from the standard port ½-20UNF-2A. This part of the reflected signal is constant, and it does not depend on the properties of the measured liquid. For measurement, the part of the signal reflected from the tip of the waveguide between 52.5 μs and 54.5 μs has been used. The trailing waves that arise due to the waveguide geometry were determined experimentally and are travelling behind the wave of interest (Figure 13). For measurements from the whole complicated shape signal, the part reflected from the waveguide tip was selected using a rectangular window in the time domain. This window in Figure 13 is shown by the dashed vertical lines. The time duration of the signal part used in measurements was determined experimentally and was *t*_m_ = 2 μs. This part of the signal depends only on the parameters of the different measured liquids and is used for density measurements.

The amplitude of the signal *U*_rw_ reflected from the distilled water at known temperature was found from the signal spectrum at the frequency *f*_0_. The reflection coefficient *R*_3_(*f*_0_) at this frequency should be minimum. In this case, the obtained *U*_rw_(*f*_0_) was used as the reference value according to Equation (6). All other density measurements are made with respect to the density measurement in the distilled water. It should be mentioned that this procedure enables reducing measurement errors due to the drift of electronic units of the measurement system.

**Figure 14:**
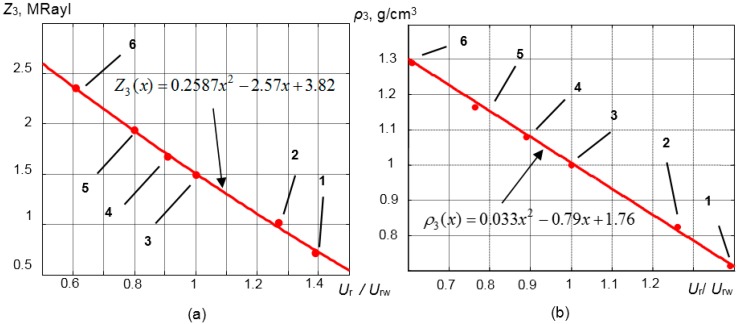
(**a**) Relationship between the normalized amplitude *U*_r_/*U*_r*w*_ and the acoustic impedance of the measured liquid; (**b**) relationship between the normalized amplitude *U*_r_/*U*_rw_ and the density of the measured liquid. (1, ethyl ether; 2, ethyl alcohol; 3, distilled water; 4, 20% sugar/water; 5, 40% sugar/water; 6, 60% sugar/water).

The experimental setup of the density measurement technique was calibrated according to the algorithm proposed by us, which consists of the following steps:

- The reflected signals *U*_r_(*T*), *U*_rw_(*T*) and the signals *U*_tr_(*T*), *U*_trw_(*T*) transmitted through the distilled water and the liquid used for calibration are recorded.
- The time delay τ_3_(*T*) in the liquid is measured in the transmission mode, and the ultrasound longitudinal wave velocity *c*_3_(*T*) in the liquid used for calibration is determined:
  (11) $$c_{3}(T)=\frac{l_{k}}{\tau_{3}(T)}$$
- The ratio of the reflected *U*_r_(*T*), *U*_rw_(*T*) signals’ spectra, normalized with respect to distilled water, is calculated. The signal reflected from the distilled water *U*_rw_(*T*) is used as the reference signal. Let us denote:
  (12) $$x={\left[\frac{FFT(U_{r}(T))}{FFT(U_{rw}(T))}\right]}_{|f=f_{0}}$$
  where *FFT* is the fast Fourier transform.
- Then, the acoustic impedance of the liquid *Z*_3_ is approximated by polynomial:
  (13) $$Z_{3}(x)=ax^{2}+bx+c$$
  where *a*, *b*, *c* are the coefficients depending on the materials of the waveguide and the matching layer.
- The calibration function is found (Figure 14):
  (14) $$\frac{U_{r}}{U_{rw}}=F(Z_{3})$$
  where the acoustic impedance of the liquid *Z*_3_ is given by:
  (15) $$Z_{3}(T)=\rho_{3}(x)\cdot c_{3}(T)$$

The relationships between the normalized amplitude *U*_r_/*U*_rw_, the acoustic impedance and the density of the measured liquid *Z*_3_ are presented in Figure 14a,b.

The calibration function in this case is Z_3_(*x*) = 0.2587*x*^2^ − 2.57*x* + 3.82. The relationship between the normalized amplitude *U*_r_/*U*_rw_ and the density ρ_3_ was determined exploiting Equation (8) (Figure 14b). In our case, it is ρ_3_ = 0.033*x*^2^ − 0.79*x* + 1.76. The approximation in this case was performed by the least squares approximation method from the measurement results.

The determined calibration functions are used as the reference functions when the density measurements in various liquids are performed at a room temperature. All measurements in other liquids are made with respect to the density of the distilled water.

At higher temperatures, the determined calibration functions can be also used if the corrected value of the reflected signal *U_r_* is inserted. Our experiments have shown that when the temperature *T* is increasing, the amplitude of the signal *U_r_* is decreasing for two reasons. The first one is due to a reduction of the electro-mechanical transformation of the piezoelectric element; the second one is due to temperature gradients along the waveguide. The influence of those factors may be reduced by performing measurements of the signals reflected from the tip of the waveguide in air at room temperature *U*_20°_ and at high temperatures *U_HT_*. The ratio of those amplitudes yields a correction coefficient $K_{HT}=\frac{U_{20{}^{\circ}}}{U_{HT}}$, which is used to get the corrected amplitude of the signal reflected at high temperatures:

(16) $$U_{r}^{\prime }=U_{HT}/K_{HT}$$

where $U_{r}^{\prime }$ is the corrected amplitude value, which is later used for density estimation using the above-presented calibration functions.

### 3.2. Density Measurements in Extreme Conditions

The developed ultrasonic technique was tested in-line on a Davis-Standard BC single-screw extruder with four heating blocks. For density measurement in the liquid polymer melt, the pulse-echo and the through-transmission techniques were used. The pair of ultrasonic waveguide transducers made of steel with the aluminum powder λ/4 matching layer was installed into the adaptor of the extruder. The excitation signal with the amplitude *U* = 200 V was generated by the pulse-type generator. The transmitting transducer was excited by a bipolar burst of 10 cycles.

The temperature of the liquid polymer melt during experiments was measured by a thermocouple. The pressure was measured by the pressure sensor PT 422A-10M-6/18. The pressure during the experiments was up to *p =* 0.65 MPa. The ultrasonic measurements were performed at frequency *f* = 3.3 MHz.

The signals were visualized on the oscilloscope Tektronics TDS 220. During experiments, the temperature was changed in the range *T* = 180–220 °C. Even when the tip of the waveguide was heated up to the *T* = 220 °C, the temperature of the opposite end of the waveguide to which the transducer was bonded did not exceed *T* = 90 °C. Variations of the polymer density were monitored during *t* = 100 min. The measurement system was connected to a personal computer with the digitizing board ATS9462 (16 bit, 100 MHz) for data processing.

**Figure 15:**
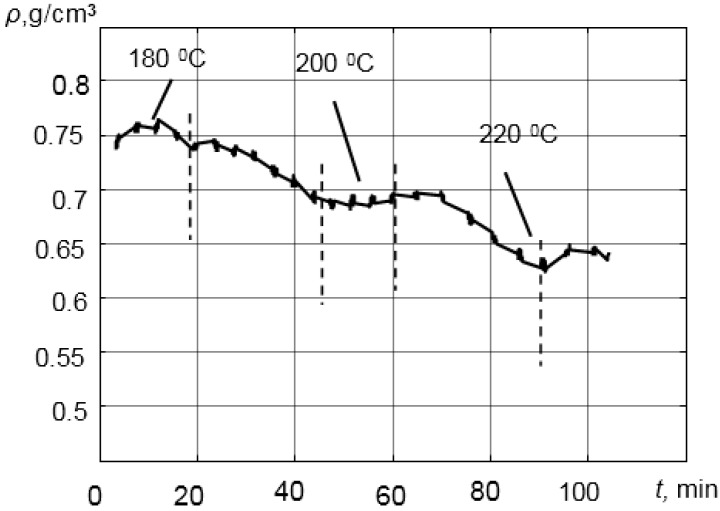
Variations of the density of the virgin polypropylene melt at different temperatures.

The results of the experiments are presented in Figure 15. Please note that temperature significantly influences the density of polypropylene (PP). The fluctuations of the density estimation were caused by the temperature changes and were caused deliberately in order to generate the density changes during the manufacturing process. In our case, the density of the PP changed in the range ρ = 0.64–0.76 g/cm^3^. The number of the measurements was *N* = 64. When the temperature is decreasing, the density of polymer melt PP is increasing. The density measurement results were very close to the values given in the literature [43].

### 3.3. Estimation of Measurement Uncertainties

In order to determine the accuracy of the proposed measurement method, analyses of the measurement uncertainty and corresponding uncertainty sources were performed. Measurement uncertainty and probability distributions of uncertainty sources were evaluated according to the BIPM (Bureau International des Poids et Mesures) guide to the expression of uncertainty in measurement [44]. The results of the metrological evaluation are presented in Table 4. Estimation of B-type uncertainty is based on past experience, taken from a handbook, extracted from a calibration report, *etc.* The B-type evaluation of the standard uncertainty shows that the measurement uncertainty can be caused by the following sources:

- Quantization of the signals.
- Electrical noise of the preamplifier and other electrical circuits.
- Variations of the temperature Δ*T*_M_ in the λ/4 matching layer.
- Variations of the temperature Δ*T*_W_ in the waveguide.

The influence of quantization of the signals and electrical noise was neglected due to relatively small values.

The A-type evaluation of the standard uncertainty is obtained from a statistical analysis of a series of independent observations that have been made under the same measurement conditions. The A-type uncertainty of the ultrasonic liquid density measurement is given by:

(17) $$S_{n}(\rho )=\sqrt{\frac{1}{n\cdot \left(n-1\right)}\sum_{i=1}^{n}(\bar{\rho_{3}{\left(T\right)}_{i}}-\rho_{3}(T}{)}_{i}{)}^{2}$$

where *S*_n_(ρ) is the experimental standard deviation of the mean value, *n* is the number of the measurements (*n* = 64) at the constant temperature *T* = 200 °C and ρ_3_ is the measured density value. Then, *S*_n_(ρ) = 3.6 × 10^−3^ g/cm^3^.

The combined standard uncertainty *U*_ρ_ of the density estimation is given by the composition of the components obtained from both A-type and B-type evaluations:

(18) $$U_{\rho }=\sqrt{u{\left(\Delta T_{M}\right)}^{2}+u{\left(\Delta T_{W}\right)}^{2}+u{\left(S_{n}(\rho )\right)}^{2}}$$

The expanded uncertainty is obtained by multiplying the combined standard uncertainty by a coverage factor *k*. To encompass approximately 95% of the possible values of the measurand, the coverage factor *k* is close to two. In this case, the expanded uncertainty is given by:

(19) $$U_{\Sigma }=k\cdot U_{\rho }$$

The expanded uncertainty *U*_Σ_ = 7.4 × 10^−3^ g/cm^3^. The sources of the measurement uncertainties and their properties are presented in Table 4.

**Table 4:** Sources of the measurement uncertainty and their properties.

Source of the uncertainty	Type	Probability Distribution/ Divisor of the Distribution	Standard Uncertainty, g/cm^3^, 10^−3^
Variation of the temperature in the λ/4 matching layer Δ*T*_M_	B	Rectangular/$\sqrt{3}$	0.3
Variation of the temperature in the waveguide Δ*T*_W_	B	Rectangular/$\sqrt{3}$	1.0
The amplitude measurement method *S*_n_(ρ)	A	σ *	3.6
Combined standard uncertainty *U*ρ		Rectangular/$\sqrt{3}$	3.7
Expanded uncertainty *U*_Σ_		Rectangular/$\sqrt{3}$	7.4

* Standard deviation.

## 4. Conclusions

This work presents a novel ultrasonic measurement technique for in-line density measurements of various liquids in extreme conditions. The main idea of this technique is the application of the λ/4 matching layer, which enhances the sensitivity of the measurement system significantly. In the case of the steel waveguide with the aluminum powder λ/4 matching layer, the dependencies of the reflection coefficient *R*_3_ increase about five times after the matching layer implementation. For density measurements in extreme conditions, the steel waveguide with the pressed aluminum powder matching layer is used. In order to avoid the influence of multiple trailing waves on measurement accuracy, the selection of the informative part of the signals in the time domain is proposed.

The density measurement technique was calibrated and verified using the reference liquids with various densities.

The experiments proved the suitability of the proposed technique for in-line density measurements in extreme conditions in a wide range of densities. The complex analysis of the measurement uncertainty has shown that the relative expanded measurement uncertainty of the polymer melt density is *U*_Σ_ = 7.4 × 10^−3^ g/cm^3^, e.g., ≈1%.
